# Unusual form of the distal bone defect of ulna with neurofibromatosis type 1: A case report

**DOI:** 10.1097/MD.0000000000017226

**Published:** 2019-10-04

**Authors:** Yiguo Shen, Fangfang Chen

**Affiliations:** a Department of Orthopedics, Zhejiang University School of Medicine Children's Hospital; b Department of Orthopedics, Zhejiang University School of Medicine Sir Run Run Shaw Hospital, Hangzhou, Zhejiang, China.

**Keywords:** bone defect, neurofibromatosis type 1, osteolysis, X-ray

## Abstract

**Rationale:**

Bone malformation occurs in 10% to 25% neurofibromatosis type 1 (NF-1) patients, and the manifestations are scoliosis, congenital arch and pseudo-joint formation, bone cyst, and pathologic fracture. However, a large segmental defect without obvious signs of bone destruction has rarely been reported.

**Patient concerns:**

A 4.5-year-old male presented with a 4-year history of shortening of the right upper limb and radial head dislocation. The X-ray indicated a lack of the distal part of the right ulna and radial head dislocation.

**Diagnosis:**

The X-ray showed obvious bone resorption at the right ulna distal, proximal stubble, and distal part of the epiphyseal residue, which was 4.3 mm shorter after 14 months. The patient was finally diagnosed with NF-1 according to the pathologic examination.

**Interventions:**

The treatment included tumor resection, ulnar osteotomy, and fixation by an Ilizarov frame.

**Outcomes:**

The Ilizarov frame was removed after 2.7 months of surgery. The radial head was successfully repositioned, and the elbow joint function was significantly improved. No recurrence of the deformity was noted until now.

**Lessons:**

Osteolysis (defect without bone destruction) is an extremely rare symptom in patients with NF1. Therefore, it is essential to make the right diagnosis by comprehensive and careful physical examination.

## 1 Introduction

Neurofibromatosis type 1 (NF-1) is an autosomal-dominant genetic disease that might involve multiple systems, including the skin, eyes, brain, and skeletal system. NF-1 occurs in approximately one in 3500 individuals.^[1]^ A total of 10% to 25% of NF-1 will present with bone malformation, which includes scoliosis, congenital arch and pseudo-joint formation, bone cyst, thinning of cortical bone, and subperiosteal bone hyperplasia.^[2]^ X-rays often show trabecula disruption, cortical distortion, sclerotic zone, bone hyperplasia, and soft tissue swelling. Herein, we report a rare case in which X-ray showed a large segmental defect without obvious signs of bone destruction.

## 2 Case presentation

**Figure 1:**
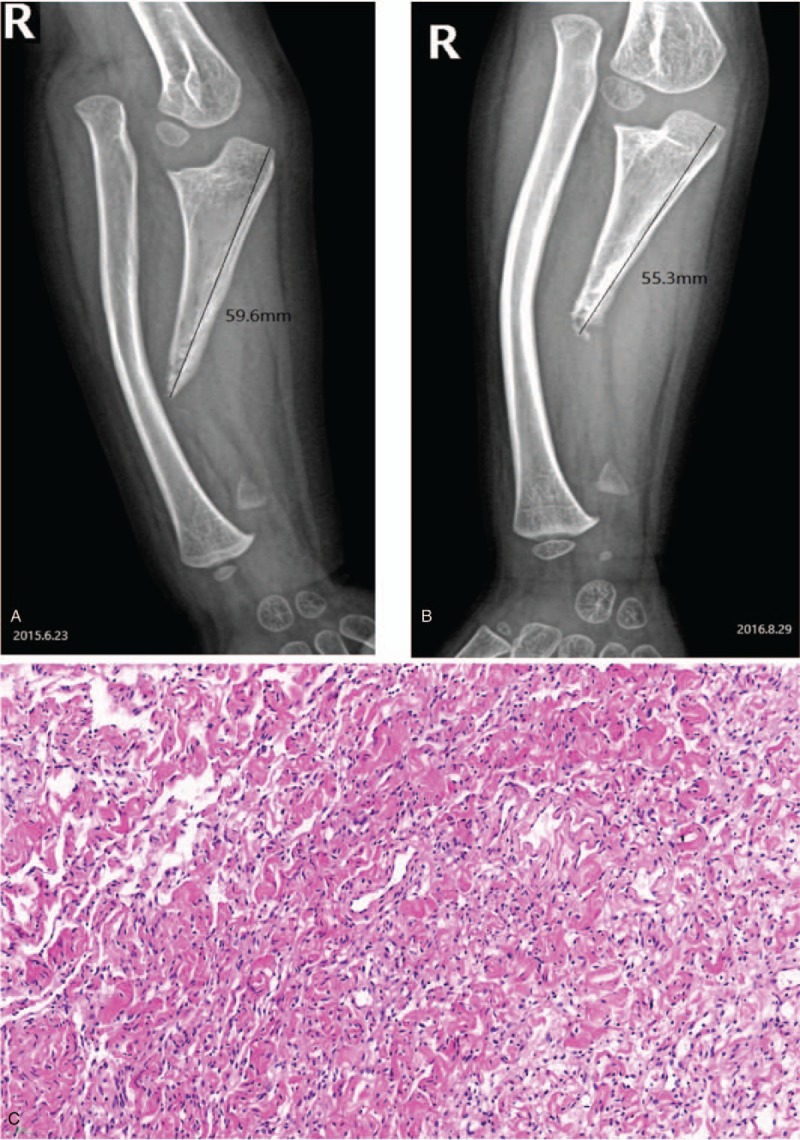
(A) X-ray imaging shows a right ulna distal defect, distal epiphyseal residual, and right radial head dislocation (2015-6-23). (B) Bone osteolysis and resorption at the right ulna distal, which was 4.3 mm shorter than that shown in (A) (2016-8-29). (C) Histopathological examination showing spindle-shaped cells with narrow nuclei interspersed with thick bands of collagen (hematoxylin and eosin staining; magnification ×40).

A 4.5-year-old boy with a 4-year history of shortening of the right upper limb was admitted to our hospital on August 29, 2016. Approximately 4 years ago, the right upper limb of the patient was shorter than the contralateral side, and physical examination did not reveal any other abnormalities. The results of routine laboratory tests were within the normal range. X-ray indicated the absence of the distal right ulna. The physicians recommended regular follow-up. On June 23, 2015, the patient was admitted to our hospital for the first time. The X-ray indicated a lack of the distal part of the right ulna and radial head dislocation (Fig. 1), but physical examinations failed to detect cafe-au-lait spots. One year later, the patient was readmitted to our hospital for exacerbated deformity. Physical examination indicated cafe-au-lait spots over the body, ranging in size, with the largest being approximately 1.5 cm. The right upper limb was approximately 2 cm shorter than the contralateral side without obvious swelling or pain. The radial head dislocation was fixed above the lateral condyle of the humerus, and the elbow flexion and extension were obviously limited. Blood tests, erythrocyte sedimentation rates, and rheumatoid factors were negative. The X-ray revealed obvious bone resorption at the right ulna distal, proximal stubble, and distal part of the epiphyseal residue. More importantly, the bone was 4.3 mm shorter than previously observed (Fig. 1). The past medical history was unremarkable and showed no history of traumatic fractures. His parents denied any family history of similar or other skin lesions.

**Figure 2:**
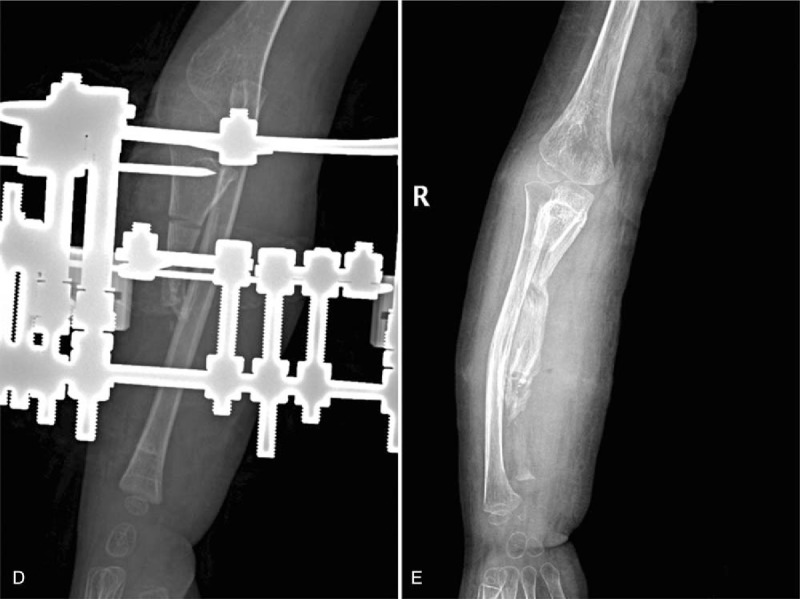
(D) The frame comprised 2 complete rings. Ulnar osteotomy was performed between the 2 rings. Lengthening was started after 7 days to lengthen the ulna and pull the radius down. (E) Following ulnar lengthening at 2 months postoperatively, the patient showed a spontaneous reduction in the radial head and the correction of forearm deformity.

Local lesion resection of the right ulnar was performed. The pathological examination was consistent with the preoperative diagnosis of neurofibroma (Fig. 1). Combined with the cafe-au-lait spots and pathological findings, a diagnosis of NF-1 was established. Then, the patient underwent a tumor resection, ulnar osteotomy, and fixation by Ilizarov frame (Fig. 2).

Lengthening was started after 7 days to lengthen the ulna and reduce the radial head. The distraction was stopped once the radial head showed reduction, and the patient was followed up every 2 weeks until a full consolidation of the callus was achieved. At 2.7 months after the surgery, the Ilizarov frame was removed (Fig. 2). The range of elbow motion improved: flexion increased from 125° to 160° and extension increased to 5°. During a 24-month follow-up after the operation, the condition of the patient remained stable and the motion of the forearm was retained.

## 3 Discussion

NF-1 is an autosomal dominant disease caused by spontaneous or inherited mutations of the NF-1 gene in chromosome region 17q11.2, which encodes neurofibromin protein.^[3]^

People with NF-1 can present with skeletal abnormalities, including osteopenia, scoliosis, sphenoid wing dysplasia, congenital tibial dysplasia, and pseudarthrosis.^[4]^ Long bone osteopathy occurs in approximately 1% to 4% of NF1 patients, while its occurrence in the general population is approximately 1 in 140,000.^[1,2,5,6]^ Radiographic findings may include cystic lesions, bony sclerosis, and bowing or thinning of the cortex. In this case, the radiology only showed a lack of the distal part of the right ulna and radial head dislocation. Compared with previous reports, this case was characterized by an aggressive osteolytic reaction without periosteum, cortical bone expansion, or bending changes. To our knowledge, this effect has not been reported before. Neurofibromatous osteopathy is one of the most challenging conditions for pediatric orthopedic surgeons, and many operations will be required for successful treatment.^[7]^ Currently, most patients are treated by excising the lesions, stabilizing the fragments with internal or external fixation, and bone grafting. In addition to this basic approach, some surgeons advocate the local administration of bone morphogenetic protein,^[8,9]^ periosteal grafting,^[10]^ and vascularized fibular grafts.^[11]^ In our case, the patient underwent tumor resection and osteotomy of ulna lengthening by Ilizarov frame. No recurrence was noted during the follow-up period.

There is a great deal of controversy surrounding the timing of surgery, the method, and duration of fixation,^[12]^ and the reason why NF-1 can affect skeletal system is unclear. Rhodes and Yang^[13]^ summarized clinical and murine studies and indicated that the NF-1 (Nf-1) gene dose is vital to the pathogenesis of NF-1-associated skeletal progenitor cells. Also, osteoclasts play a critical role in potentiating osteolytic activity, cooperating with Nf-1-deficient mesenchymal cells and osteoblasts to engender multiple NF-1-associated osseous defects, including osteoporosis, in transgenic mouse models closely recapitulating human disease. Poyrazoğlu et al^[14]^ also revealed that the majority of NF-1 patients would suffer more bone mass loss. Additionally, bone mineral metabolism was greatly changed in patients with NF-1. These findings may identify novel, targeted therapies to effectively treat skeletal anomalies due to NF-1.

Osteolysis is an extremely rare symptom in patients with NF-1. In particular, when X-rays show only bone defects without bone distortion, fracture, or destruction, misdiagnosis is inevitable. Therefore, a comprehensive and careful physical examination is essential for a correct diagnosis.

## Author contributions

**Investigation:** Fangfang Chen.

**Writing – original draft:** Fangfang Chen.

**Writing – review & editing:** Yiguo Shen.

Yiguo Shen orcid: 0000-0002-2119-0662.
